# Trauma is a public health issue

**DOI:** 10.1080/20008198.2017.1375338

**Published:** 2017-10-09

**Authors:** Kathryn M. Magruder, Katie A. McLaughlin, Diane L. Elmore Borbon

**Affiliations:** ^a^ Department of Psychiatry and Behavioral Sciences, Medical University of South Carolina, Charleston, S.C, U.S.A; ^b^ Department of Psychology, University of Washington, Seattle, WA, U.S.A; ^c^ Department of Psychiatry and Behavioral Sciences, UCLA-Duke University National Center for Child Traumatic Stress, Washington, D.C, U.S.A

**Keywords:** Trauma, public health, prevention, early intervention, traumatic stress, disaster, Trauma, salud pública, prevención, intervención temprana, estrés traumático, desastre, 创伤, 公共健康, 预防, 早期干预, 创伤应激, 灾难, • Exposure to trauma is pervasive in societies worldwide and is associated with substantial costs to the individual and society, making it a significant global public health concern.• We present factors at individual, relationship, community, and society levels—as explanatory factors in both the occurrence of trauma and its sequelae.• We highlight targets for prevention of trauma and early intervention at all of these levels.• We describe the essential role of public health policies in addressing trauma as a global public health issue.

## Abstract

Exposure to trauma is pervasive in societies worldwide and is associated with substantial costs to the individual and society, making it a significant global public health concern. We present evidence for trauma as a public health issue by highlighting the role of characteristics operating at multiple levels of influence – individual, relationship, community, and society – as explanatory factors in both the occurrence of trauma and its sequelae. Within the context of this multi-level framework, we highlight targets for prevention of trauma and its downstream consequences and provide examples of where public health approaches to prevention have met with success. Finally, we describe the essential role of public health policies in addressing trauma as a global public health issue, including key challenges for global mental health and next steps for developing and implementing a trauma-informed public health policy agenda. A public health framework is critical for understanding risk and protective factors for trauma and its aftermath operating at multiple levels of influence and generating opportunities for prevention.

## Abstract (es)

La exposición al trauma es general en las sociedades de todo el mundo y está asociada con costos sustanciales para el individuo y la sociedad, lo que la convierte en un importante problema mundial de salud pública. Presentamos evidencia del trauma como problema de salud pública al destacar el papel de las características que operan en múltiples niveles de influencia -individuo, relación, comunidad y sociedad- como factores que explican tanto la ocurrencia del trauma como sus secuelas. En el contexto de este marco multi-nivel, destacamos objetivos para la prevención del trauma y sus consecuencias posteriores, y ofrecemos ejemplos de los casos en que los abordajes preventivos de salud pública han tenido éxito. Por último, describimos el papel esencial de las políticas de salud pública a la hora de abordar el trauma como problema de salud pública mundial, incluyendo los retos clave para la salud mental global y los próximos pasos para desarrollar e implementar una agenda de políticas de salud pública. Un marco de salud pública es fundamental para comprender los factores de riesgo y de protección para el trauma y sus secuelas, que operan en múltiples niveles de influencia y generan oportunidades de prevención.

## Abstract (zh)

摘要：创伤暴露在世界范围的社会中都普遍存在，且对个人和社会造成了重大的损失。这使得创伤暴露成为了一个显著的世界公共健康问题。我们强调有特色的应对（characteristics operating）在多重水平上（个人、关系、社区和社会）的影响是创伤的发生及其后果的解释因素，从而提出创伤是公共健康问题的证据。在这一多水平框架的背景下，我们强调了预防创伤的目标和随之带来的后果，并举出利用公共健康方法成功预防创伤的例子。最终，我们描述了公共卫生政策在解决作为全球公共健康问题的创伤时扮演的重要角色，全球精神健康面临的关键挑战，以及发展和实施针对创伤的公共健康政策日程的未来步骤。不论是理解创伤的危险和保护因素以及创伤后应对方式在多重水平上的影响，还是促发预防机会，公共健康框架都至关重要。

## 1. Public health impact of trauma

Exposure to trauma is pervasive in societies worldwide. Population-based data from various countries indicate that a majority of adults will experience a traumatic event at some point in their lives, despite cross-national variation in the prevalence of specific types of traumatic events (Benjet et al., 2016; Burri & Maercker, 2014). Trauma exposure is also common in children and adolescents around the world. A substantial proportion of children globally are exposed to trauma as a result of armed conflict, natural disasters, and other humanitarian emergencies (World Health Organization, 2013b). An estimated 230 million children currently live in countries impacted by armed conflicts (UNICEF, 2014), which increases risk of experiencing displacement, witnessing violence and death, and being orphaned, kidnapped, raped, or recruited as child soldiers (UNICEF, 2009).

Traumatic events do not only occur at random, but can be influenced by individual characteristics, peer group relationships, community characteristics, and socio-political factors. At the individual level, for example, the likelihood of experiencing particular types of trauma varies by sex, age, race/ethnicity, and sexual orientation (Garcia-Moreno, Jansen, Ellsberg, Heise, & Watts, 2006; Kessler, Sonnega, Bromet, Hughes, & Nelson, 1995; McLaughlin et al., 2013; Rees et al., 2011). Community and socio-political factors also influence the likelihood of trauma occurrence across geographic locations. Certain types of traumas (e.g. violence) are more likely to occur in certain locations (e.g. metropolitan areas and conflict zones) (McLaughlin et al., 2013; Perkonigg, Kessler, Storz, & Wittchen, 2000). Moreover, different communities will have diverse trauma recovery trajectories based on their pre-trauma community characteristics (Nakagawa & Shaw, 2004). Thus, it is important not to overlook these characteristics in considering both trauma exposure and outcome.

The public health impact of trauma exposure is staggering for both communities and individuals. Catastrophic events such as natural and man-made disasters and terrorist attacks can have devastating effects on the social fabric of society and communities, not only involving injuries and loss of life, but also related to property destruction and infrastructure damage. This aftermath, coupled with high levels of resulting migration, can create prolonged disruption in the delivery of social services and the dissolution of social support networks. These community-level consequences can persist for lengthy periods, often fundamentally changing the physical and social landscape of a community (Galea et al., 2002; Hollifield et al., 2008; Rosenbaum, 2006). Low and middle income countries (LMICs) are disproportionately affected. Collective violence (e.g. war, genocide) is 10 times more common in LMICs versus high income countries (HICs) (World Health Organization, 2002), and LMICs carry the brunt of migration problems caused by disasters and violence. In 2015, 65.3 million people were forcibly displaced, the vast majority from LMICs. According to the United Nations High Commission for Refugees (UNHCR), the top hosting countries are Turkey (2.5 million), Pakistan (1.6 million), Lebanon (1.1 million), and Iran (1.0 million).

Exposure to trauma is particularly detrimental when it occurs in childhood or adolescence, disrupting numerous aspects of development in cognitive, emotional, and social domains, leading to adverse mental health and educational outcomes (Cicchetti & Toth, 1995; Koenen, Moffit, Caspi, Taylor, & Purcell, 2003) with long-term consequences for learning and memory (Teicher, Anderson, & Polcari, 2012), emotional functioning (De Bellis et al., 1994; McCrory et al., 2011; McLaughlin & Hatzenbuehler, 2009; Pollak & Sinha, 2002; Pollak, Vardi, Putzer Bechner, & Curtin, 2005), social relationships, elevated risk of re-victimization (Cole & Putnam, 1992; DiLillo, 2001; Follette, Polusney, Bechtle, & Naugle, 1996), and mental disorders (Kilpatrick et al., 2003; McLaughlin et al., 2012, 2013).

Post-traumatic stress disorder (PTSD), which is inextricably linked with trauma, is in itself a profound public health burden. Individuals who develop PTSD following trauma experience impaired role functioning and reduced life course opportunities (Kessler, 2000). PTSD was associated with high levels of disability, and in developing countries disability associated with PTSD was higher than most common medical conditions except for headaches and chronic pain (Ormel et al., 2008). The economic costs of PTSD are staggering, with work impairment associated with the disorder estimated at 3.6 days per month per person with PTSD. The annual lost productivity due to PTSD is estimated at over $3 billion dollars in the U.S. alone (Kessler, 2000).

Because the development of PTSD is conditional on trauma exposure, PTSD may be the most preventable of mental disorders. We have the unique opportunity to reduce the population burden of PTSD both by preventing trauma exposure and by delivering timely interventions in the wake of trauma to those most at risk. The following sections explore these opportunities based on a public health approach, extending previous work (Magruder, Kassam-Adams, Thoresen, & Olff, 2016) by expanding examples and policy implications.

## 2. Public health model of traumatic stress

**Figure 1.**
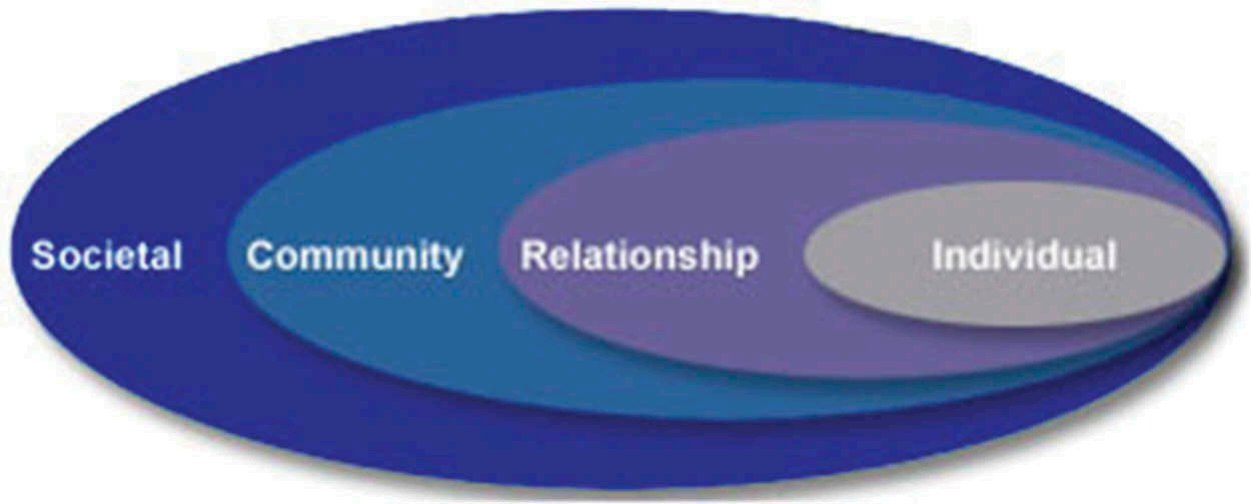
The social-ecological model, a framework for prevention from the U.S. centers for disease control and prevention (Dahlberg & Krug, 2002).

Current approaches to public health are explicitly multi-level and concerned with identifying *causes* of health states (Krieger, 1994; Susser, 1998) with the ultimate goal of preventing disease onset. With problems that have a behavioural component, a public health model encompasses factors operating at multiple levels of influence, such as family, school, and cultural levels (see Figure 1) (Bronfenbrenner, 1979) (Dahlberg & Krug, 2002).

In the case of trauma-related problems, the important components are the trauma itself, those who are exposed to trauma, their relationships, the variety of environmental factors playing a role in shaping the likelihood of both trauma exposure and outcome, and societal factors, attitudes, and characteristics that influence trauma likelihood and intervention. Such a multi-level approach provides a public health framework for developing an array of strategies aimed at preventing the occurrence and sequelae of trauma. Furthermore, these levels can be considered as points of intervention and opportunities for prevention. The classic prevention framework includes three levels: primary; secondary; and tertiary (Commission on Chronic Illness, 1957). The aim of primary prevention is to prevent the actual occurrence of the disease or illness. The purpose of secondary prevention is to intervene early in the disease process for cure or optimal outcomes. Tertiary prevention is aimed at preventing the disability that accompanies an illness or disease. Each of these levels of prevention can be implemented at different system levels, including society at large, the community, the family, and the individual. Subsequently, risk and protective factors can be translated into multi-sectoral, multi-modal, and multi-level preventive interventions (De Jong, 2010; Wiist et al., 2014). Table 1 provides a few examples of prevention strategies at each level.

**Table 1.** Prevention examples within a public health framework.

Prevention level			
Social-ecological level	Primary	Secondary	Tertiary
*Individual*	Alcohol education programmes for young adults to prevent high risk drinking, thus reducing risk for physical and sexual assaults	Minimization of ongoing stressors for trauma-exposed individuals to prevent onset of full PTSD	Effective and timely treatment of PTSD to prevent development of comorbidities
*Relationship*	Programmes to prevent bullying in schools	Early intervention for trauma-exposed children	Training for foster parents of children with psychological problems related to trauma
*Community*	Lighting parking lots, streets, and campuses to prevent crime	Development and promotion of proactive community preparedness measures to anticipate response to disasters	Education programmes to promote understanding of psychological sequelae of trauma exposure and to reduce stigma
*Societal*	Policies to limit firearm possession	Policies to promote and facilitate early intervention for trauma victims	Peace agreements to prevent political violence

From a public health perspective, preventing exposure to trauma is an obvious strategy, and such efforts can be targeted to all levels of the social-ecological model. A number of strategies are aimed at reducing the likelihood of trauma exposure in individuals. Alcohol education programmes for young adults, such as those implemented on U.S. college campuses, can reduce high-risk drinking which in turn may reduce exposure to traumatic events like physical and sexual assaults, accidents, or motor vehicle accidents (Katz & Moore, 2013). At the relationship level, there are programmes to strengthen the abilities and sensitivity of family caregivers as well as programmes to prevent bullying in schools. At the community level, examples include lighting parking lots, streets, and campuses to prevent crime. Even design of highways to reduce traffic accidents can be seen as an environmental response to reduce motor vehicle accidents and thus reduce trauma occurrence. Other examples include neighbourhood watch programmes to prevent crime and community members preventing sexual assaults in refugee camps. At the societal level, some traumas can be prevented by promoting appropriate social norms. For example, policy changes in Australia have been successful at reducing firearms deaths and injuries (Chapman, Alpers, Agho, & Jones, 2006). Because alcohol consumption is price sensitive, especially for those under 21 years old in the U.S., changes in pricing of alcohol have been proposed as a means of reducing hazardous drinking, and thereby reducing alcohol-related traumatic events, such as family violence and crimes (Chaloupka, 1993; Chaloupka, Grossman, & Saffer, 1998; Presley, Meilman, & Leichliter, 2002). Many targets are not thought of as trauma prevention even though they serve as such, for example, increased screening at airports and at major events (e.g. World Cup). Even seemingly unrelated approaches such as improving education levels, eradicating poverty, and decreasing social inequality may have positive primary prevention outcomes because these factors are important predictors of health, mental health, and human rights (Daar et al., 2007).

From some of these examples, it is clear that efforts aimed at preventing trauma may have multiple beneficial outcomes. In the case of reducing the number of motor vehicle accidents, physical trauma and death are the primary targets, and psychological sequelae are the secondary. The same is true of the United Nations’ (UN) efforts to decrease political violence, to install war tribunals and prosecute perpetrators, and to stimulate efforts to have international laws which condemn human rights violations or ban landmines, or similarly when governments attempt to prevent mass terrorist attacks or the re-emergence of violence. Disaster preparedness training can also have a preventive effect, such as setting quality standards for buildings in earthquake- or landslide-prone areas or river beds, setting higher quality standards for the construction of nuclear power stations, providing better access to land in areas with landslides, creating better alarm systems for floods, cyclones, or hurricanes, and providing sheltered areas and evacuation plans in regions that are hit by volcano eruptions or typhoons (De Jong, 2011).

Secondary prevention can also be directed at various levels. Early intervention with those who have been trauma exposed and are symptomatic focuses on individuals within various settings and environments (rather than focusing on environments per se). There is evidence that for those exposed to trauma and disasters, ongoing non-traumatic stressors are also predictive of PTSD onset and course (as well as for other problems, such as alcohol use disorders) (Cerdá et al., 2013, 2014). Thus, minimization of these ongoing stressors may prevent the onset of PTSD and other psychiatric disorders. In fact, humanitarian relief operations aimed at large populations often focus on shelter, food, water, sanitation, and physical disease control. Evidence suggests that individual-level secondary prevention interventions aimed at bolstering resilience and reducing the likelihood of adverse effects following trauma are effective. For example, the military in several countries have developed pre-deployment programmes to prevent PTSD (Hourani, Council, Hubal, & Strange, 2011), and a recent meta-analysis suggests that intervention within one month can be effective in children and adolescents following a single trauma exposure (Kramer & Landolt, 2011). At the relationship level, examples include shelters for survivors of domestic violence, interventions aimed at couples where domestic violence has already occurred, and foster care for children who are abused or neglected and unable to live with their parents or another relative. Some secondary prevention approaches focus on communities. For example, community support, as in the case of vigils for survivors and their families following disasters or mass violence, is often seen as helpful. Safety considerations, such as providing food and shelter and reuniting families post-disaster, may go a long way to reassure disaster survivors and prevent the development of mental health symptoms. Such support and intervention does not occur successfully in poorly organized or disenfranchised communities; thus, efforts to develop and promote strong proactive communities and build capacity to respond to disasters can also be seen as secondary prevention (Laborde, Magruder, Caye, & Parrish, 2013). From the societal point of view, policies that promote early intervention are also helpful as secondary prevention measures.

With the aim of preventing the progression of disease and disability, most tertiary prevention programmes are squarely in the clinical arena and are seen as part of standard treatment for PTSD or other trauma-related mental health problems; however, there are some examples of societal level tertiary prevention. For example, in the international arena tertiary prevention may aim at peace-keeping and peace-enforcing troops, as well as peace agreements to prevent the reemergence of political violence. Lower rates of PTSD have been found in states that have legislated legal protections for lesbian, gay, bisexual, and transgendered individuals as compared to states that do not have protective legislation (Hatzenbuehler, McLaughlin, Keyes, & Hasin, 2010). Similarly, promotion of reconciliation and mediation skills between groups on the community level may be seen as tertiary prevention (De Jong, 2011). On the family and personal level, most tertiary prevention programmes aim at preventing the progression of disease and disability, and are seen as part of standard treatment for PTSD or other trauma-related mental health problems.

Recasting traumatic stress treatment as an approach to prevent the development of comorbidities (e.g. depression and substance use disorders) and to improve functioning (even if not eliminating symptoms entirely) may open the door for novel clinical approaches. For example, the use of support dogs may improve functioning and reduce disability by enabling someone with traumatic stress to enjoy greater societal participation – even if symptoms are not completely alleviated (Krause-Parello, Sami, & Padden, 2016). At the relationship level, there is evidence that training foster parents of children with significant psychological problems resulting from trauma exposure may help to prevent the development of additional problems (Leve et al., 2012). As with secondary prevention, both community and societal support can also be helpful. They can help to establish service availability and to promote use of services by reducing stigma.

## 3. The essential role of public health policies

Effective public policies can help to shape societal norms concerning public health issues and are ideally informed by an evidence base. Such policies have been critical in securing the 10 great public health achievements of the twenty-first century, including tobacco control, motor vehicle safety, and prevention and control of infectious diseases (Centers for Disease Control and Prevention, 2011). Policies must focus on preventing traumatic events, when possible, providing early intervention services for survivor communities at risk of poor post-trauma outcomes, and reducing stigma. There are currently several overarching mental health policy challenges facing communities and countries plagued by violence and trauma. Because trauma and mental health issues are inextricably linked, they also share many challenges.

Mental health issues (and thus trauma-related issues) have largely been ignored as a priority in the global public health agenda (Saracena et al., 2007; Saxena & Skeen, 2012). For example, the UN Millennium Development Goals (MDGs), a set of development targets agreed to by the international community, failed to include any specific focus on mental health or trauma (Saxena & Skeen, 2012). The main focus of the recent World Health Organization (WHO) Global Action Plan for the Prevention and Control of NCDs is on four specific types – cardiovascular diseases, cancer, chronic respiratory diseases, and diabetes – and on four shared behavioural risk factors – tobacco use, unhealthy diet, physical inactivity, and harmful use of alcohol. Among the other conditions of public health importance that are explicitly mentioned in the plan are mental disorders, violence, and injuries (World Health Organization, 2013a). This indicates that mental health is beginning to gain impetus alongside other leading public health policy priorities; however, until full parity exists for mental health concerns, the issue of preventing trauma and intervening early in its aftermath will fail to receive sufficient global attention as a policy priority.

Much work remains ahead to understand the nature and treatment of mental disorders in global context. Greater investment is needed in global mental health research in high, middle, and low income countries. Cross-national population-based initiatives, such as the World Mental Health Surveys, can play an important role in understanding the prevalence, impact, and health systems’ response to mental disorders (Patel, 2012). More research is needed to comprehend the broad physical health, mental health, and developmental impacts of childhood polyvictimization (Felitti et al., 1998; Finkelhor, Ormrod, & Turner, 2007). Additional priorities for improving global mental health research include a focus on adequately training researchers around the world, ensuring a bi-directional flow of information and partnerships in the global mental health research community (Patel & Prince, 2010). Recommendations for building global trauma research capacity include: providing quality training and distance learning opportunities, supporting international fellowships, promoting memberships in collaborative research teams, improving accessibility of the scientific literature, and encouraging researchers to share their knowledge with policymakers and key stakeholders (Fodor et al., 2014). Unlike other service sectors that rely heavily on equipment or supplies, mental health services are primarily dependent on human resources (World Health Organization, 2011). Many have identified shortcomings of the current global mental health workforce, including limitations on the number and types of workers trained in mental health care, perhaps due to poor working conditions and low status associated with the mental health professions (Saracena et al., 2007).

According to WHO, an immediate and sizeable investment is needed to scale up a well-trained global mental health workforce. One method suggested for expanding the workforce is task shifting, or redistribution of tasks from highly qualified health workers to health workers with shorter training and fewer qualifications (World Health Organization, 2008). An example of a task shifting approach is WHO’s Problem Management Plus, a low-intensity intervention for adults with symptoms of common mental health problems. This intervention uses lay helpers supervised by skilled mental health professionals in communities exposed to adversity (Dawson et al., 2015). In addition to more general mental health workforce strategies, several important efforts are underway by leaders in the trauma field to develop trauma-focused competencies to help mental health professionals build foundational trauma knowledge and skills (Bisson et al., 2010; Cook, Newman, & The New Haven Trauma Competency Group, 2014; Layne et al., 2014).

In many countries, a wide gap exists between the need for mental health services and the availability of treatment (World Health Organization, 2011). This is particularly challenging for LMIC, which are home to over 80% of the global population, but utilize less than 20% of the mental health resources (Saxena, Thornicroft, Knapp, & Whiteford, 2007). Worldwide there exists a troubling cycle of disadvantage, social exclusion, and mental disorders. The consequent treatment gap is a contravention of basic human rights, as more than 75% of those identified with serious anxiety, mood, impulse control, or substance use disorders in the World Mental Health surveys in LMIC received no care at all, despite substantial role disability. Further, mental health resources are often inequitably distributed among countries, regions, and within communities. Such inequities can occur in access to care, use and outcomes of care, and by geographic region, race/ethnicity, gender, sexual orientation, and socioeconomic status (Ngui, Khasakhala, Ndetei, & Roberts, 2010). Governments must invest more of their health budgets toward these inequities in order to adequately address mental health and trauma-focused prevention and early intervention.

Much attention around the world has focused on the benefits of integrating mental health care into primary care and other settings where people receive services (Ngui et al., 2010; Patel & Prince, 2010; World Health Organization, 2001). Such integration of physical and mental health care is especially important for trauma-exposed populations, as they often seek help in primary care rather than mental health settings (U.S. Department of Veterans Affairs, 2002). While much of the global population is only seen in primary care, mental health issues (such as PTSD) often go undiagnosed in this setting (Üstün & Sartorius, 1995). Further, physical disease is often accompanied by psychological morbidity that is not always recognized by primary care providers (World Health Organization, 2001). The fact that medical comorbidities often develop with PTSD (and other psychiatric disorders) is yet another reason that primary care clinicians need training on the recognition and treatment of mental health conditions.

Many efforts are underway to integrate physical and mental health and provide trauma-informed training to health care providers, including in the Department of Veterans Affairs in the U.S. and a variety of European countries. This includes a policy of routine screening by health care providers for trauma exposure and resources to assist providers in addressing trauma and PTSD in primary care (U.S. Department of Veterans Affairs, 2002). Similar practices and policies are underway in other systems around the world; however, integration of mental health and trauma-informed services remains the exception and not the rule in many communities. Many suggest that a true public health approach requires mental health integration beyond primary care to include sectors such as education, justice, welfare, and labour through partnerships with government, non-governmental organizations, and the faith-based community (Collins, Insel, Chockalingam, Daar, & Maddox, 2013; Ko et al., 2008),

Stigma associated with mental health issues, such as traumatic stress, can serve as a barrier to mental health treatment and positive outcomes. According to the WHO (World Health Organization, 2010), those with mental health conditions are the most marginalized and vulnerable groups in society and may face restrictions in exercising their political and civil rights. In addition, they can have difficulty accessing health care, social services, and educational and employment opportunities. Efforts to address stigma and discrimination related to mental health issues are underway. Among the strategies used include social activism, public education, and contact with persons with mental illness. A recent meta-analysis of outcome studies revealed that both education and contact had positive effects on reducing stigma for adults and adolescents with a mental illness (Corrigan, Morris, Michaels, Rafacz, & Rusch, 2012).

## 4. Summary

A public health framework is critical for understanding risk and protective factors for trauma and its aftermath operating at multiple levels of influence and generating opportunities for prevention at each of these levels. Primary prevention efforts should be aimed at preventing exposure to trauma itself. Secondary prevention should be directed at the prevention of trauma-related sequelae, in particular post-traumatic stress disorder. Tertiary prevention should slow the progression of trauma-related illness and disability. Advantages to adopting a public health approach to trauma include involvement of families, communities, and policymakers – making them more informed, activated, and supportive of prevention and early intervention efforts. The task is not easy, as trauma is a general term that encompasses a variety of experiences that range from rape to earthquakes. Preventive efforts will be vastly different for different traumas; thus, public health trauma advocates may need to form strategic alliances with unlikely partners, for example, highway safety officials concerned with traffic accidents, university officials concerned with sexual assaults, or government officials in areas at high risk for natural disasters. Furthermore, given the global nature of trauma, approaches may need to adapted for different cultures. Despite these challenges, there have been some modest successes in implementing mental health and trauma policies around the world. Building on these successes will help to establish a public health approach to trauma.

## References

[CIT0001] Benjet C., Bromet E., Karam E. G., Kessler R. C., McLaughlin K. A., Ruscio A. M., … Koenen K. C. (2016). The epidemiology of traumatic event exposure worldwide: Results from the World Mental Health Survey Consortium. *Psychological Medicine*, 46(2), 327–9. doi:10.1017/s0033291715001981 26511595 PMC4869975

[CIT0002] Bisson J. I., Tavakoly B., Witteveen A. B., Ajdukovic D., Jehel L., Johansen V. J., … Olff M. (2010). TENTS guidelines: Development of post-disaster psychosocial care guidelines through a delphi process. *British Journal of Psychiatry*, 196, 69–74. doi:10.1192/bjp.bp.109.066266 20044665

[CIT0003] Bronfenbrenner U. (1979). *The ecology of human development: Experiments by nature and design*. Cambridge, MA: Harvard University Press.

[CIT0004] Burri A., & Maercker A. (2014). Differences in prevalence rates of PTSD in various European countries explained by war exposure, other trauma and cultural value orientation. *BMC Research Notes*, 7(1), 407. doi:10.1186/1756-0500-7-407 24972489 PMC4114166

[CIT0005] Centers for Disease Control and Prevention (2011). Ten great public health achievements: United States 2001-2010. *Morbidity and Mortality Weekly Report*, 60, 619–623. 21597455

[CIT0006] Cerdá M., Bordelois P. M., Galea S., Norris F. H., Tracy M., & Koenen K. C. (2013). The course of posttraumatic stress symptoms and functional impairment following a disaster: What is the lasting influence of acute versus ongoing traumatic events and stressors? *Social Psychiatry and Psychiatric Epidemiology*, 48, 385–395. doi:10.1007/s00127-012-0560-3 22878832 PMC3504624

[CIT0007] Cerdá M., Richards C., Cohen G. H., Calabrese J. R., Liberzon I., Tamburrino M., … Koenen K. C. (2014). Civilian stressors associated with alcohol use disorders in the national guard. *American Journal of Preventive Medicine*, 47, 461–466. doi:10.1016/j.amepre.2014.06.015 25089013 PMC4171186

[CIT0008] Chaloupka F. J. (1993). Effects of price on alcohol-related problems. *Alcohol Health Research World*, 17, 46–53. PMC6683806 12154648

[CIT0009] Chaloupka F. J., Grossman M., & Saffer H. (1998). The effects of price on the consequences of alcohol use and abuse In Galanter M. (Ed.), *Recent developments in alcoholism, Vol 14: The consequences of alcoholism* (pp. 331–346). New York: Plenum Press. 10.1007/0-306-47148-5_15 9751952

[CIT0010] Chapman S., Alpers P., Agho K., & Jones M. (2006). Australia’s 1996 gun law reforms: Faster falls in firearm deaths, firearm suicides, and a decade without mass shootings. *Injury Prevention*, 12, 365–372. doi:10.1136/ip.2006.013714 17170183 PMC2704353

[CIT0011] Cicchetti D., & Toth T. L. (1995). A developmental psychopathology perspective on child abuse and neglect. *Journal of the American Academy of Child & Adolescent Psychiatry*, 34, 541–565. doi:10.1097/00004583-199505000-00008 7775351

[CIT0012] Cole P. M., & Putnam F. W. (1992). Effect of incest on self and social functioning: A developmental psychopathology perspective. *Journal of Consulting and Clinical Psychology*, 60, 174–184. 1592946 10.1037//0022-006x.60.2.174

[CIT0013] Collins P. Y., Insel T. R., Chockalingam A., Daar A., & Maddox Y. T. (2013). Grand challenges in global mental health: Integration in research, policy and practice. *Plos Medicine*, 10(4), e1001434. doi:10.1371/journal.pmed.1001434 23637578 PMC3640093

[CIT0014] Commission on Chronic Illness (1957). *Chronic illness in the USA* (Vol. 1). Cambridge, MA: Harvard University Press.

[CIT0015] Cook J. M., Newman E., & The New Haven Trauma Competency Group (2014). A consensus statement on trauma mental health: The new haven competency conference process and major findings. *Psychological Trauma: Theory, Research, Practice, and Policy*, 6, 300–307. doi:10.1037/a0036747

[CIT0016] Corrigan P. W., Morris S. B., Michaels P. J., Rafacz J. D., & Rusch N. (2012). Challenging the public stigma of mental illness: A meta-analysis of outcome studies. *Psychiatric Services*, 63, 963–973. doi:10.1176/appi.ps.201100529 23032675

[CIT0017] Daar A. S., Singer P. A., Persad D. L., Pramming S. K., Matthews D. R., Beaglehole R., … Bell J. (2007). Grand challenges in chronic non-communicable diseases. *Nature*, 450, 494–496. doi:10.1038/450494a 18033288

[CIT0018] Dahlberg L. L., & Krug E. G. (2002). Violence: A global public health problem In Krug E., Dahlberg L. L., Mercy J. A., & Lozano R. (Eds.), *World report on violence and health* (pp. 1–56). Geneva, Switzerland: World Health Organization.

[CIT0019] Dawson K. S., Bryant R. A., Harper M., KuoweiTay A., Rahman A., Schafer A., & Van Ommeren M. (2015). Problem management plus (PM+): A WHO transdiagnostic psychological intervention for common mental health problems. *World Psychiatry*, 14(3), 354–357. doi:10.1002/wps.20255 26407793 PMC4592660

[CIT0020] De Bellis M. D., Chrousos G. P., Dorn L. D., Burke L., Helmers K., Kling M. A., & Trickett P. K. (1994). Hypothalamic-pituitary-adrenal axis dysregulation in sexually abused girls. *Journal of Clinical Endocrinology and Metabolism*, 78, 249–255. doi:10.1210/jcem.78.2.8106608 8106608

[CIT0021] De Jong J. T. (2010). A public health framework to translate risk factors related to political violence and war into multilevel preventive interventions. *Social Science & Medicine*, 70, 71–79. doi:10.1016/j.socscimed.2009.09.044 19883967

[CIT0022] De Jong J. T. (2011). Disaster public mental health In Stein D. J., Friedman M., & Blanco C. (Eds.), *Post-traumatic stress disorder* (pp. 217–262). Oxford: John Wiley and Sons.

[CIT0023] DiLillo D. (2001). Interpersonal functioning among women reporting a history of childhood sexual abuse: Empirical findings and methodological issues. *Clinical Psychology Review*, 21, 553–576. 11413867 10.1016/s0272-7358(99)00072-0

[CIT0024] Felitti V. J., Anda R. F., Nordenberg D., Williamson D. F., Spitz A. M., Edwards V., … Marks J. S. (1998). Relationship of childhood abuse and household dysfunction to many of the leading causes of death in adults: The Adverse Childhood Experiences (ACE) Study. *American Journal of Preventive Medicine*, 14, 245–258. 9635069 10.1016/s0749-3797(98)00017-8

[CIT0025] Finkelhor D., Ormrod R. K., & Turner H. A. (2007). Poly-victimization: A neglected component in child victimization. *Child Abuse & Neglect*, 31, 7–26. doi:10.1016/j.chiabu.2006.06.008 17224181

[CIT0026] Fodor K. E., Unterhitzenberger J., Chou C. Y., Kartal D., Leistner S., Milosavljevic M., … Alisic E. (2014). Is traumatic stress research global? A bibliometric analysis. *European Journal of Psychotraumatology*, 5, 23269. doi:10.3402/ejpt.v5.23269 PMC3930940 24563730

[CIT0027] Follette V., Polusney M. A., Bechtle A. E., & Naugle A. E. (1996). Cumulative trauma: The impact of child sexual abuse, adult sexual assault, and spouse abuse. *Journal of Traumatic Stress*, 9, 25–35. 8750449 10.1007/BF02116831

[CIT0028] Galea S., Ahern J., Resnick H., Kilpatrick D. G., Bucuvalas M., Gold J., & Vlahov D. (2002). Psychological sequelae of the September 11 terrorist attacks in New York city. *New England Journal of Medicine*, 346, 982–987. doi:10.1056/NEJMsa013404 11919308

[CIT0029] Garcia-Moreno C., Jansen H. A. F. M., Ellsberg M., Heise L., & Watts C. (2006). WHO Multi-country study on women’s health and domestic violence against women. *Lancet*, 368, 1260–1269. doi:10.1016/S0140-6736(06)69523-8 17027732

[CIT0030] Hatzenbuehler M. L., McLaughlin K. A., Keyes K. M., & Hasin D. S. (2010). The impact of discriminatory laws on psychiatric disorders in LGB populations: A prospective study. *American Journal of Public Health*, 100, 452–459. doi:10.2105/AJPH.2009.168815 20075314 PMC2820062

[CIT0031] Hollifield M., Hewage C., Gunawardena C. N., Piyadasa K., Bopagoda K., & Weerarathnege K. (2008). Symptoms and coping in Sri Lanka 20-21 months after the 2004 tsunami. *British Journal of Psychiatry*, 192, 39–44. doi:10.1192/bjp.bp.107.038422 18174508

[CIT0032] Hourani L. L., Council C. L., Hubal R. C., & Strange L. B. (2011). Approaches to the primary prevention of posttraumatic stress disorder in the military: A review of the stress control literature. *Military Medicine*, 176, 721–730. 22128712 10.7205/milmed-d-09-00227

[CIT0033] Katz J., & Moore J. (2013). Bystander education training for campus sexual assault prevention: An initial meta-analysis. *Violence and Victims*, 28, 1054–1067. 24547680 10.1891/0886-6708.vv-d-12-00113

[CIT0034] Kessler R. C. (2000). Posttraumatic stress disorder: The burden to the individual and to society. *Journal of Clinical Psychiatry*, 61(suppl. 5), 4–12. 10761674

[CIT0035] Kessler R. C., Sonnega A., Bromet E., Hughes M., & Nelson C. B. (1995). Posttraumatic stress disorder in the National Comorbidity Survey. *Archives of General Psychiatry*, 52, 1048–1060. 7492257 10.1001/archpsyc.1995.03950240066012

[CIT0036] Kilpatrick D. G., Ruggiero K. J., Acierno R., Saunders B. E., Resnick H. S., & Best C. L. (2003). Violence and risk of PTSD, major depression, substance abuse/dependence, and comorbidity: Results from the National Survey of Adolescents. *Journal of Consulting and Clinical Psychology*, 71, 692–700. 12924674 10.1037/0022-006x.71.4.692

[CIT0037] Ko S. J., Ford J. D., Kassam-Adams N., Berkowitz S. J., Wilson C., Wong M., … Layne C. M. (2008). Creating trauma-informed systems: Child welfare, education, first responders, health care, juvenile justice. *Professional Psychology: Research and Practice*, 39, 396–404. doi:10.1037/0735-7028.39.4.396

[CIT0038] Koenen K. C., Moffit T. E., Caspi A., Taylor A., & Purcell S. (2003). Domestic violence is associated with environmental suppression of IQ in young children. *Development and Psychopathology*, 15, 297–311. 12931829 10.1017/s0954579403000166

[CIT0039] Kramer D. N., & Landolt M. A. (2011). Characteristics and efficacy of early psychological interventions in children and adolescents after single trauma: A meta-analysis. *European Journal of Psychotraumatology*, 2. doi:10.3402/ejpt.v2i0.7858 PMC3402147 22893820

[CIT0040] Krause-Parello C. A., Sami S., & Padden E. (2016). Military veterans and canine assistance for post-traumatic stress disorder: A narrative review of the literature. *Nurse Education Today*, 47, 43–50. doi:10.1016/j.nedt.2016.04.020 27179660

[CIT0041] Krieger N. (1994). Epidemiology and the web of causation: Has anyone seen the spider? *Social Science and Medicine*, 39, 887–903. 7992123 10.1016/0277-9536(94)90202-x

[CIT0042] Laborde D. J., Magruder K. M., Caye J., & Parrish T. B. (2013). Feasibility of disaster mental health preparedness training for Black communities. *Disaster Medicine and Public Health Preparedness*, 7, 303–312. doi:10.1001/dmp.2012.18 22752411

[CIT0043] Layne C. M., Strand V., Popescu M., Kaplow J. B., Abramowitz R., Stuber M., … Pynoos R. S. (2014). Using the core curriculum on childhood trauma to strengthen clinical knowledge in evidence-based practitioners. *Journal of Clinical Child & Adolescent Psychology*, 43, 286–300. doi:10.1080/15374416.2013.865192 24484506

[CIT0044] Leve L. D., Harold G. T., Chamberlain P., Landsverk J. A., Fishe R. P. A., & Vostanis P. (2012). Practitioner review: Children in foster care–vulnerabilities and evidence-based interventions that promote resilience processes. *Journal of Child Psychology and Psychiatry,And Allied Disciplines*, 53(12), 1197–1211. doi:10.1111/j.1469-7610.2012.02594.x PMC3505234 22882015

[CIT0045] Magruder K. M., Kassam-Adams N., Thoresen S., & Olff M. (2016). Prevention and public health approaches to trauma and traumatic stress: A rationale and call to action. *European Journal of Psychotraumatology*, 7, 29715. doi:10.3402/ejpt.v7.29715 26996536 PMC4800286

[CIT0046] McCrory E. J., De Brito S. A., Sebastian C. L., Mechelli A., Bird G., Kelly P. A., & Viding E. (2011). Heightened neural reactivity to threat in child victims of family violence. *Current Biology*, 21, R947–948. doi:10.1016/j.cub.2011.10.015 22153160

[CIT0047] McLaughlin K. A., Green J. G., Gruber M. J., Sampson N. A., Zaslavsky A., & Kessler R. C. (2012). Childhood adversities and first onset of psychiatric disorders in a national sample of adolescents. *Archives of General Psychiatry*, 69, 1151–1160. doi:10.1001/archgenpsychiatry.2011.2277 23117636 PMC3490224

[CIT0048] McLaughlin K. A., & Hatzenbuehler M. L. (2009). Mechanisms linking stressful life events and mental health problems in a prospective, community-based sample of adolescents. *Journal of Adolescent Health*, 44, 153–160. doi:10.1016/j.jadohealth.2008.06.019 19167664 PMC2881598

[CIT0049] McLaughlin K. A., Koenen K. C., Hill E., Petukhova M., Sampson N. A., Zaslavsky A., & Kessler R. C. (2013). Trauma exposure and posttraumatic stress disorder in a US national sample of adolescents. *Journal of the American Academy of Child & Adolescent Psychiatry*, 52, 815–830. doi:10.1016/j.jaac.2013.05.011 23880492 PMC3724231

[CIT0050] Nakagawa Y., & Shaw R. (2004). Social capital: A missing link in disaster recovery. *International Journal of Mass Emergencies and Disasters*, 22, 5–34.

[CIT0051] Ngui E. M., Khasakhala L., Ndetei D., & Roberts L. W. (2010). Mental disorders, health inequalities and ethics: A global perspective. *International Review of Psychiatry*, 22, 235–244. doi:10.3109/09540261.2010.485273 20528652 PMC2935265

[CIT0052] Ormel J., Petukhova M., Chatterji S., Aguilar-Gaxiola S., Alonso J., Angermeyer M. C., … Kessler R. C. (2008). Disability and treatment of specific mental and physical disorders across the world. *British Journal of Psychiatry*, 192, 368–375. doi:10.1192/bjp.bp.107.039107 18450663 PMC2681238

[CIT0053] Patel V. (2012). Global mental health: From science to action. *Harvard Review of Psychiatry*, 20, 6–12. doi:10.3109/10673229.2012.649108 22335178 PMC3335087

[CIT0054] Patel V., & Prince M. (2010). Global mental health: A new global health field comes of age. *JAMA: Journal of the American Medical Association*, 303, 1976–1977. doi:10.1001/jama.2010.616 20483977 PMC3432444

[CIT0055] Perkonigg A., Kessler R. C., Storz S., & Wittchen H.-U. (2000). Traumatic events and post-traumatic stress disorder in the community: Prevalence, risk factors, and comorbidity. *Acta Psychiatrica Scandinavica*, 101, 46–59. 10674950 10.1034/j.1600-0447.2000.101001046.x

[CIT0056] Pollak S. D., & Sinha P. (2002). Effects of early experience on children’s recognition of facial displays of emotion. *Development and Psychopathology*, 38, 784–791. 10.1037//0012-1649.38.5.784 12220055

[CIT0057] Pollak S. D., Vardi S., Putzer Bechner A. M., & Curtin J. J. (2005). Physically abused children’s regulation of attention in response to hostility. *Child Development*, 76, 968–977. doi:10.1111/j.1467-8624.2005.00890.x 16149995

[CIT0058] Presley C. A., Meilman P. W., & Leichliter J. S. (2002). College factors that influence drinking. *Journal of Studies on Alcohol*, 14, 89–90. 10.15288/jsas.2002.s14.82 12022732

[CIT0059] Rees S., Silove D., Chey T., Ivancic L., Steel Z., Creamer M., … Forbes D. (2011). Lifetime prevalence of gender-based violence in women and the relationship with mental disorders and psychosocial function. *JAMA: Journal of the American Medical Association*, 306, 513–521. 21813429 10.1001/jama.2011.1098

[CIT0060] Rosenbaum S. (2006). US health policy in the aftermath of Hurricane Katrina. *JAMA: Journal of the American Medical Association*, 295, 437–440. doi:10.1001/jama.295.4.437 16434635

[CIT0061] Saracena B., van Ommeren M., Batniji R., Cohen A., Gureje O., Mahoney J., … Underhill C. (2007). Barriers to improvement of mental health services in low-income and middle-income countries. *Lancet*, 370, 1164–1174. doi:10.1016/S0140-6736(07)61263-X 17804061

[CIT0062] Saxena S., & Skeen S. (2012). No health without mental health: Challenges and opportunities in global mental health. *African Journal of Psychiatry*, 15, 397–400. 23379015

[CIT0063] Saxena S., Thornicroft G., Knapp M., & Whiteford H. (2007). Resources for mental health: Scarcity, inequity, and inefficiency. *Lancet*, 370, 878–879. doi:10.1016/S0140-6736(07)61239-2 17804062

[CIT0064] Susser M. (1998). Does risk factor epidemiology put epidemiology at risk? Peering into the future. *Journal of Epidemiology and Community Health*, 52, 608–611. 10023453 10.1136/jech.52.10.608 PMC1756623

[CIT0065] Teicher M. H., Anderson C. M., & Polcari A. (2012). Childhood maltreatment is associated with reduced volume in the hippocampal subfields CA3, dentate gyrus, and subiculum. *Proceedings of the National Academy of Sciences*, 109, E563–572. doi:10.1073/pnas.1115396109 PMC3295326 22331913

[CIT0066] U.S. Department of Veterans Affairs (2002). Post-traumatic stress disorder: Implications for primary care. Retrieved from http://www.publichealth.va.gov/docs/vhi/posttraumatic.pdf 23487872

[CIT0067] UNICEF (2009). *Machel study 10-year strategic review: Children and conflict in a changing world*. New York: Office of the Special Representative of the Secretary-General for Children, & Armed Conflict.

[CIT0068] UNICEF (2014). With 15 million children caught up in major conflicts, UNICEF declares 2014 a devastating year for children [Press release] Retrieved from http://www.unicef.org/media/media_78058.html?p=printme

[CIT0069] Üstün T. B., & Sartorius N. (1995). *Mental illness in general health care: An international study*. West Sussex, England: Wiley and Sons.

[CIT0070] Wiist W. H., Barker K., Arya N., Rohde J., Donohoe M., White S., … Hagopian A. (2014). The role of public health in the prevention of war: Rationale and competencies. *American Journal of Public Health*, 104, e34–e37. doi:10.2105/AJPH.2013.301778 PMC4062030 24825229

[CIT0071] World Health Organization (2001). Mental health: New understanding, new hope. Retrieved from http://www.who.int/whr/2001/en/whr01_en.pdf?ua=1

[CIT0072] Krug, E., G., Dahlberg, L. L., Mercy, J. A., Zwi, A., & Lozano, R. (Eds.). (2002). *World report on violence and health*. Geneva: World Health Organization.

[CIT0073] WHO, PEPFAR, UNAIDS. (2008). *Task shifting: Rational redistribution of tasks among health workforce teams. Global recommendations and guidelines*. Geneva: World Health Organization.

[CIT0074] World Health Organization (2010). Mental health and development: Targeting people with mental health conditions as a vulnerable group. Retrieved from http://whqlibdoc.who.int/publications/2010/9789241563949_eng.pdf?ua=1

[CIT0075] World Health Organization (2011). Human resources for mental health: Workforce shortages in low- and middle-income countries. Retrieved from http://whqlibdoc.who.int/publications/2011/9789241501019_eng.pdf?ua=1

[CIT0076] World Health Organization (2013a). *Global action plan for the prevention and control of noncommunicable diseases 2013-2020*. Geneva: World Health Organization.

[CIT0077] World Health Organization (2013b). *Mental health action plan 2013-2020*. Geneva: World Health Organization.

